# Evaluation of key aroma compounds and protein secondary structure in the roasted Tan mutton during the traditional charcoal process

**DOI:** 10.3389/fnut.2022.1003126

**Published:** 2022-10-18

**Authors:** Yong-Rui Wang, Song-Lei Wang, Rui-Ming Luo

**Affiliations:** ^1^College of Agriculture, Ningxia University, Yinchuan, China; ^2^College of Food and Wine, Ningxia University, Yinchuan, China

**Keywords:** roasted Tan mutton, key aroma compounds, OAVS, aroma recombination experiment, protein secondary structure, correlation analysis

## Abstract

The traditional charcoal technique was used to determine the changes in the key aroma compounds of Tan mutton during the roasting process. The results showed that the samples at the different roasting time were distinguished using GC-MS in combination with PLS-DA. A total of 26 volatile compounds were identified, among which 14 compounds, including (*E*)-2-octenal, 1-heptanol, hexanal, 1-hexanol, heptanal, 1-octen-3-ol, 1-pentanol, (*E*)-2-nonenal, octanal, 2-undecenal, nonanal, pentanal, 2-pentylfuran and 2-methypyrazine, were confirmed as key aroma compounds through the odor activity values (OAV) and aroma recombination experiments. The OAV and contribution rate of the 14 key aroma compounds were maintained at high levels, and nonanal had the highest OAV (322.34) and contribution rate (27.74%) in the samples after roasting for 10 min. The content of α-helix significantly decreased (*P* < 0.05), while the β-sheet content significantly increased (*P* < 0.05) during the roasting process. The content of random coils significantly increased in the samples roasted for 0–8 min (*P* < 0.05), and then no obvious change was observed. At the same time, β-turn content had no obvious change. Correlation analysis showed that the 14 key aroma compounds were all positively correlated with the content of α-helix and negatively correlated with the contents of β-sheet and random coil, and also positively correlated with the content of β-turn, except hexanal and 2-methypyrazine. The results are helpful to promoting the industrialization of roasted Tan mutton.

## Introduction

Regardless of religious restrictions, mutton is extensively consumed owing to its great nutritional value, including iron, zinc, high-quality protein, fat and vitamins (1). The consumption of mutton in China, the world's largest mutton production and consumption country, has steadily increased (2). Tan sheep, one of the most well-known and popular sheep in Ningxia Hui Autonomous Region of China, have frequently boarded the official dinner to entertain international guests (3). The unique geographical environment, forage grass and water quality of the Ningxia region result in a low “off-flavor” of Tan mutton.

Roasted mutton, including roasted sheep leg and mutton shashlik, is a classic and handy meal that is popular across the world (4). During the roasting process of mutton, the reactions, like Maillard reaction, lipid oxidation and the interactions between the components of the meat produce a large number of aroma compounds (5). The aroma compounds, including aliphatic aldehydes, ketones, alcohols, acids and esters, were usually generated by lipid degradation, and these compounds were responsible for the animal species-specific meat aroma (6, 7). The heterocyclic compounds, like nitrogen-containing and oxygen-containing, as well as sulfur-containing chemicals, are regularly produced by the Maillard process and contribute to the fundamental aroma of meat (8). Furthermore, the interaction between the Maillard reaction and the lipid degradation also plays a significant role in the generation of the cooked meat aroma (9). However, the overall aroma of Tan mutton can be altered by modifying the manufacturing conditions, and temperature is an important factor in this process (10).

In the recent 40 years, the key aroma compounds in many types of meat were evaluated by GC-MS combined with GC-O and odor active values (OAV) (8). Based on OAV, a series of compounds, such as hexanal, 1-octen-3-ol, nonanal and octanal, were confirmed as the primary aroma compounds in roasted mutton using a typical charcoal roasting method (11). Different roasting methods, including microwave, electricity and superheated steam, also produced similar aroma compounds in roasted mutton (12–14). However, to our knowledge, little has been known about the changes in aroma compounds or odor expression and its alterations during the roasting process. It is thus necessary to understand the relationship between the changes and key control factors of aroma compounds in roasted mutton, thereby helping producers control the aroma of products (15).

Protein can combine with aroma compounds (16). When the protein's capacity to attach to the aromatic compounds changes, it impacts the retention of the aroma compounds in the product, which has a substantial impact on the product's aroma perception (17). Polypeptides' original conformation was broken during the roasting process, resulting in increased thermal motion, loss of secondary and tertiary structure, and rupture of intermolecular forces, such as electrostatic or non-polar interactions and disulfide bonds (18–20). As a result, the meat's sensory quality after cooking, like tenderness and aroma, was affected by the denaturation of proteins and changes in fiber structure resulting from heat treatment (21, 22). The appropriateness of meat for cooking is determined mostly by macroscopical factors. However, microstructure changes in meat after cooking are the basis and causes of these macroscopical alterations, which have received little attention so far (23).

Thus, the study aimed to (i) select the key aroma compounds by GC-O and study the changes of these compounds in the composition and concentration using GC-MS in roasted Tan mutton during the traditional charcoal process; (ii) identify the key aroma compounds in roasted Tan mutton using OAV and aroma recombination tests; (iii) study the changes in the protein secondary structure of roasted Tan mutton during the roasting process; (iv) determine correlation between the protein secondary structure and key aroma compounds in roasted Tan mutton.

## Materials and methods

### Materials

The Tan mutton used in this experiment was randomly obtained from the hind legs of Yanchi Tan sheep (30 ± 1 kg, 9 months age) from Ningxia Xinhai Food Co., Ltd. (Yanchi, China). The following chemicals were purchased from Sigma-Aldrich (Shanghai, China): 1,2-dichlorobenzene (internal standard, 99.7%) and n-alkanes (C_5_-C_32_, 98%), hexanal (95%), heptanal (97%), 1-heptanol (97%), benzaldehyde (99.5%), 2-methypyrazine (99%), (*E*)-2-undecenal (96%), pentanal (99%), octanal (99%), 1-hexanol (99%), 1-pentanol (99%), (*E*)-2-octenal (97%), nonanal (99.5%), (*E*)-2-nonenal (97%), 2-pentylfuran and 1-octen-3-ol (98%). Methanol (analytical grade) was purchased from Thermo Fisher Scientific Co., Ltd. (Shanghai, China).

### Sample preparation

The bones were removed from the leg of the Tan sheep, and then the mutton washed with tap water to clean the blood and other impurities on the surface (24). After washing, the mutton was cut into small pieces (1.5 × 1.5 × 1.0 cm). Then eight pieces of Tan mutton were put into iron sticks, and then placed on the fire for grilling. The Tan mutton was roasted on a barbeque grill 5 cm from the charcoal fire at 250–270°C, flipping over the clusters every 20 s. The water content of tan mutton decreased from (raw meat) 69.34–72.02 to 48.33–53.25% (roasting for 14 min). The sensory assessment findings revealed that 10 min was the optimal roasting period for the mutton, and the mutton roasted for 10 min had the best flavor and overall acceptability with the unanimous agreement of panelists. The core and surface temperatures of the roasted Tan mutton at 10 min were 79.5–81.2 and 85.6–95.7°C, respectively. The samples were roasted for 0, 2, 4, 6, 8, 10, 12, and 14 min. Three replicate samples (eight iron sticks each) were prepared and subjected to the following analyses.

### Aroma analysis

#### GC-MS analysis

Aroma analysis of roasted Tan mutton was performed by a GC-MS system (GC-MS 2010 plus, SHIMADZU, Japan) jointed with solid-phase microextraction (SPME) fiber. Briefly, 2 ± 0.01 g of the sample after crushed were placed into a 15 mL headspace bottle with 4 μL of internal standard (1,2-dichlorobenzene, 6.42 μg/mL in methanol). After mixing with a vortex, the headspace bottle was sealed with a PTFE diaphragm, and placed in a water bath at 55°C for 20 min. The aged SPME fiber (50/30 μm DVB / CAR / PDMS) was inserted into a sealed extraction bottle and kept on the top of the mutton sample for adsorption for 30 min, and then transferred to the GC inlet for desorption at 250°C for 5 min. The chromatographic capillary column was DB-WAX (30 m × 0.25 mm × 0.25 μm, Agilent Technologies, Santa Clara, CA). The GC and MS were carried out in accordance with our previous study (25).

#### GC-O analysis

A GC system equipped with an olfactory detection port (GC 2014, SHIMADZU, Japan) and a DB-WAX column (60 m × 0.25 mm × 0.25 μm; Agilent Technologies, Inc.) was used for GC-O analysis The GC conditions were the same as for the GC-MS. The effluent was divided in a 1:1 ratio between the MS and the sniffer port. This sniffing experiment was conducted by three professional appraisers. They were required to keep track of the retention time according to the time of the stopwatch and describe the aroma characteristics as soon as they appear in the sniffing port.

#### Identification and quantitation of aroma compounds

The aroma compounds of roasted Tan mutton were identified using a mass spectrometry library (MS), linear retention indices (LRI) and odor characteristics (O) in comparison to authentic flavor standards (S) (Table 1). The retention of a homologous sequence of n-alkanes (C_5_-C_32_) was used to compute the LRI. The odorants were validated by comparing the retention time and ion fragments of samples with authentic flavor standards in GC-MS analysis under similar chromatographic conditions. As an internal standard, 1,2-dichlorobenzene was used to semiquantify the aroma compounds. In particular, a 5-point calibration curve was used to measure the quantities of odorants (OAV >1) in an odorless mutton model. The odorless mutton model, in a nutshell, consisted of an odorless mutton matrix, realistic flavor criteria and ultrapure water. The odorless mutton matrix was made according to previous studies (4, 26), with some modifications. Briefly, diethyl ether and n-pentane were added to the mutton (diethyl ether–n-pentane–mutton puree ratio of 2:1:1, m/m/m). After shaking for 12 h, the organic solvent was extracted 5 times. The samples were then frozen in an FD-1A-50 freeze-dryer using liquid nitrogen (Shanghai Zheng-Qiao Science Instrument Plant, Ltd., Beijing, China) at −50°C for 24 h. Sensory panelists assessed the aroma characteristics of roasted mutton and the recombination model.

**Table 1:** Identification of aroma compounds in roasted Tan mutton for 0–14 min.

**No**.	**Volatile compounds^a^**	**Threshold (μg/kg)^b^**	**RI**		**Odor description**	**ID^e^**
			**Literature^c^**	**Calculated^d^**		
1	1-Heptanol	5.4	1462	1473	floral	MS,O,RI,S
2	1-Hexanol	5.6	1359	1351	green, fruity, oily	MS,O,RI,S
3	1-Nonanol	46	1673	1770	rose, citrus	MS,O,RI
4	1-Octanol	120	1573	1558	fatty, waxy	MS,O,RI
5	1-Octen-3-ol	1	1456	1462	mushroom	MS,O,RI,S
6	1-Pentanol	150	1274	1275	sweet, balsamic	MS,O,RI,S
7	2,3-Butanediol	/	1583	1584	green	MS,O,RI
8	(*E*)-2-Heptenal	40	1291	1286	herbaceous, green, oily	MS,O,RI
9	(*E*)-2-Nonenal	0.19	1517	1514	green, fatty, tallowy	MS,O,RI,S
10	(*E*)-2-Octenal	3	1396	1392	sweet, fatty, mild	MS,O,RI,S
11	(*E*)-2-Undecenal	0.78	1755	1754	green, fruity, fatty	MS,O,RI,S
12	Hexanal	4.5	1064	1068	floral, fruity, fatty	MS,O,RI,S
13	Octanal	0.59	1273	1277	fruity, nutty, oily	MS,O,RI,S
14	Heptanal	2.8	1163	1160	herbaceous, green, oily	MS,O,RI,S
15	Benzaldehyde	750	1534	1528	bitter almond, aromatic, popcorn	MS,O,RI
16	Nonanal	1.1	1369	1367	sweet melon	MS,O,RI,S
17	Pentanal	12	964	967	woody, fatty	MS,O,RI,S
18	Acetic acid	99	1401	1398	sour, pungent, strong	MS,O,RI
19	Hexanoic acid	890	1854	1857	lamby, oily	MS,O,RI
20	Decanoic acid	10	2281	2269	rancid, oily	MS,O,RI
21	Nonanoic acid	6.8	2174	2173	rancid, oily, fatty	MS,O,RI
22	Octanoic acid	3	2067	2075	cheesy, waxy	MS,O,RI
23	Hexanoic acid methyl ester	77	1189	1192	fruity	MS,O,RI
24	6-Methyl-5-hepten-2-one	68	1342	1361	roasted peanuts, tallowy	MS,O,RI
25	2-Pentylfuran	6	1215	1228	vegetable, earthy	MS,O,RI,S
26	2-Methypyrazine	30	1238	1243	roasted, meaty	MS,O,RI,S

^a^The aroma compounds in roasted mutton. ^b^Aroma thresholds obtained from reference Sohail et al. (8). ^c^Data in literature. ^d^Data determined according to the retention time of n-alkanes (C_5_-C_32_). ^e^Identification analysis. MS, mass spectrometry; LRI, linear retention indices; O, odor qualities; S, authentic flavor standards.

### Protein secondary structure analysis

The protein's secondary structure of roasted Tan mutton was determined using attenuated total reflection (ATR) by Fourier transform infrared spectroscopy (FTIR; Bruker, Germany). Roasted mutton was vacuum-frozen (freezing temperature −25°C, cold trap temperature −30°C, vacuum degree 20 Pa), dried for 48 h and then crushed into 200 mesh powder. Then 2 mg sample of roasted mutton were added with 100 mg KBr, placed in a mortar to grind and crush until evenness, and then pressed into thin slices for analysis by mid-infrared spectroscopy. The instrument's parameters included absorbance spectra ranging from 500 to 4000 cm^−1^, a resolution of 4 cm^−1^, and a scan rate of 100 times. The protein secondary structure information was contained in the amide I spectrum in the mid-IR spectral range of 1700–1600 cm^−1^, which was caused by the expansion vibration of C = O (27). The superposition of different protein secondary structure peak components resulted in the formation of the amide I band. The second-order derivation and deconvolution technique were used to further deconstruct the peaks in the amide I band of the original protein infrared spectra that were not discernable into multiple sub-peaks. Peakfit 4.12 software was used to perform deconvolution and curve-fitting on the amide I band distribution.

### Sensory evaluation

The sensory evaluation was carried out in accordance with previous reports (28, 29). The panelists consisted of 10 graduate students (five males and five females, aged from 22 to 25) who did not suffer rhinitis and were non-smokers (30, 31). Before the experiments, ISO 4121:2003 and GB/T 29604-2013 guidelines were used to train all panelists for 30 days. Firstly, the aroma characteristics of a 54-aroma kit (Le Nez du Vin^®^, France) were distinguished and described for 20 days (once 5 days, each training lasting for 1 h). Secondly, five olfactory qualities, including meaty, fatty, roasty, grassy, and sweet, were chosen for the panelists to assess the aroma quality of roasted Tan mutton. This assessment lasted for 30 min and was performed 15 times within 30 days. Finally, these panelists were qualified to do the sensory evaluation. The sensory evaluation panel evaluated the sensory properties of roasted Tan mutton on a five-point scale (4~5: very strong, 3~4: strong, 2~3: medium, 1~2: weak, 0~1: very weak). To avoid odor interaction between samples, panelists were required to take a 30 s break during the experiment.

### Statistical analysis

Excel was used to analyze the average value and standard deviation of the data, and the data were expressed as means ± standard deviation. Duncan's multiple range test (*P* < 0.05) was used to analyze differences between individual means using SPSS 19.0 software (IBM Corporation, USA). The PCA chart of the electronic nose data was made by SIMCA14.0 software. The Pearson correlation analysis was performed by R software. The other graphs were made by Origin 18C software.

## Results

### Discrimination of roasted Tan mutton

**Figure 1:**
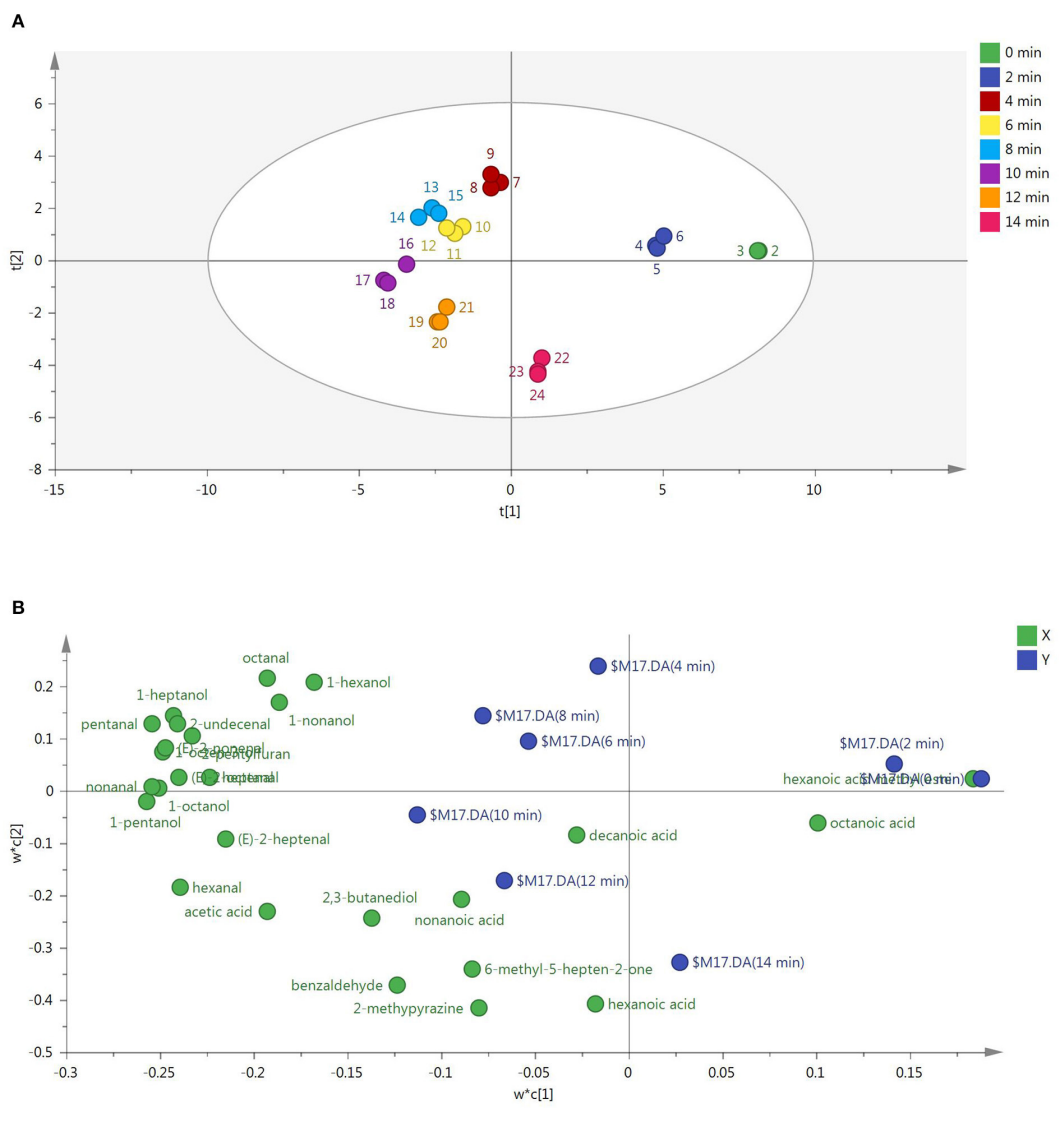
**(A)** PLS-DA of roasted mutton during the roasting process based on aroma compounds. Samples at each roasting time were measured for 3 replicates. **(B)** PLS-DA of aroma compounds in roasted mutton during the roasting process. X and Y in the top right represented roasted mutton with different roasting time and aroma compounds (4).

To discriminate the samples of roasted Tan mutton, GC-MS was coupled with PLS-DA. As shown in Figure 1A, the PLS-DA score plot showed a distinct separation of the eight sample groups. R^2^X, R^2^Y and Q^2^ values of 0.982, 0.983, and 0.951 were obtained, indicating that the built model was stable and predictive. The raw meat and sample roasted for 2 min located in the first quadrant of the PLS-DA score plot (Figure 1B), among which hexanoic acid methyl ester was the major chemical family forming the odors of raw meat. The mutton samples roasted for 4, 6, and 8 min located in the second quadrant. This distribution was heavily influenced by alcohols and aldehydes, including 1-octene-3-ol, 1-nonanol, 1-octanol, pentanal, heptanal, (*E*)-2-nonenal, (*E*)-2-octenal, otanal, 1-hexanol, 1-heptanol, nonanal, (*E*)-2-undecenal, and 2-pentylfuran. The mutton samples roasted for 10 and 12 min appeared in the third quadrant related to 1-pentanol, hexanal, (*E*)-2-heptenal, decanoic acid, nonanoic acid, hexanoic acid, acetic acid, 2,3-butanediol, 6-methyl-5-hepten-2-one, 2-methylpyrazine, and benzaldehyde. The mutton sample with roasting for 14 min appeared in the fourth quadrant related to octanoic acid.

### The changes in composition and concentrations of aroma compounds in roasted Tan mutton

As shown in Tables 1, 2, a total of 26 aroma compounds were identified using DB-WAX columns in all samples, including seven alcohols, 10 aldehydes, five acids, two heterocyclics, one ester and one ketone. In comparison, no significant changes in any of the odorants were found across the eight samples (*P* > 0.05). Aldehydes and alcohols accounted for more than 65% of all odorants. Methyl ester hexanoic acid was identified in the raw meat and disappeared after roasting for 2 min. Compared with the mutton samples roasted for 2–14 min, the concentration of octanoic acid was significantly higher (*P* < 0.05) in the raw meat. In contrast, benzaldehyde and pentanal appeared in the mutton roasted for 2 min, with a high concentration in the mutton roasted for 14 min. 1-octanol, hexanal, 1-pentanol, 2-pentylfuran, heptanal, 2,3-butanediol, (*E*)-2-heptenal, octanal, (*E*)-2-octenal, nonanal and nonanoic acid were found to have the highest concentrations in the mutton roasted for 8 and 10 min. The mutton roasted for 14 min had the highest concentrations of 6-methyl-5-hepten-2-one, acetic acid, and hexanoic acid. Interestingly, 2-methypyrazine had the highest concentration in the sample roasted for 14 min.

**Table 2:** Quantitation analysis of aroma compounds in roasted Tan mutton during the roasting process.

				**Volatile flavor compounds retention (**μ**g/kg)**							
**No**.	**Volatile compounds**	**Calibration equations**	***R* ^2^**	**0 min**	**2 min**	**4 min**	**6 min**	**8 min**	**10 min**	**12 min**	**14 min**
1	1-Heptanol	y = 0.2391x + 0.0178	0.9855	2.19 ± 0.19f	3.12 ± 0.04f	12.68 ± 1.39b	12.15 ± 0.24bc	14.18 ± 0.69a	11.17 ± 0.22cd	10.07 ± 0.42d	7.50 ± 0.12e
2	1-Hexanol	y = 0.0517x + 0.0232	0.9928	11.52 ± 0.05e	11.89 ± 0.30e	16.38 ± 0.70c	24.09 ± 1.48a	16.89 ± 0.88bc	18.42 ± 1.09b	13.95 ± 0.68d	11.00 ± 0.46e
3	1-Nonanol	—	—	0.77 ± 0.18c	0.85 ± 0.09c	2.17 ± 0.12b	2.26 ± 0.24b	2.21 ± 0.17b	2.20 ± 0.17b	3.04 ± 0.15a	ND
4	1-Octanol	—	—	4.38 ± 0.02e	14.21 ± 0.46d	24.30 ± 1.31b	19.79 ± 1.74c	27.16 ± 2.71b	32.91 ± 2.41a	26.54 ± 1.08b	18.62 ± 2.09c
5	1-Octen-3-ol	y = 0.0296x + 0.0156	0.9904	102.26 ± 0.92f	130.07 ± 5.86e	236.43 ± 9.34c	328.55 ± 24.08a	304.24 ± 4.07b	320.31 ± 3.77ab	228.43 ± 5.55cd	209.03 ± 5.64d
6	1-Pentanol	y = 0.4106x + 0.0144	0.9915	11.75 ± 0.17e	22.42 ± 1.12d	139.81 ± 3.39b	138.68 ± 7.65b	136.51 ± 5.06b	153.06 ± 4.08a	132.53 ± 2.76b	122.45 ± 2.02c
7	2,3-Butanediol	—	—	10.44 ± 0.81d	10.86 ± 0.78d	6.61 ± 0.14e	36.92 ± 2.08a	11.36 ± 1.00d	33.88 ± 1.62b	26.75 ± 1.70c	28.97 ± 1.84c
8	(*E*)-2-Heptenal	—	—	1.36 ± 0.04f	1.12 ± 0.011f	3.88 ± 0.43e	11.36 ± 0.20d	22.24 ± 1.77a	17.39 ± 0.38b	12.86 ± 0.69c	12.65 ± 0.24cd
9	(*E*)-2-Nonenal	y = 0.5312x + 0.0312	0.9832	0.30 ± 0.03c	0.21 ± 0.06c	4.16 ± 0.30a	3.90 ± 0.04a	3.92 ± 0.70a	4.03 ± 0.19a	3.11 ± 0.10b	2.59 ± 0.31b
10	(*E*)-2-Octenal	y = 0.6473x + 0.0023	0.9649	0.53 ± 0.02d	2.19 ± 0.09cd	5.29 ± 0.65b	13.65 ± 2.15a	15.14 ± 1.45a	15.21 ± 0.77a	13.86 ± 0.52a	3.97 ± 0.16bc
11	2-Undecenal	y = 0.5374x + 0.0457	0.9826	0.20 ± 0.02g	0.43 ± 0.10f	1.72 ± 0.13c	1.26 ± 0.10d	2.19 ± 0.03a	1.89 ± 0.04b	1.31 ± 0.07d	0.96 ± 0.08e
12	Hexanal	y = 0.0182x + 0.0010	0.9915	23.62 ± 0.49g	94.90 ± 2.14f	234.41 ± 6.56e	378.74 ± 8.10d	430.31 ± 3.70c	658.11 ± 13.18a	565.49 ± 5.17b	423.28 ± 11.34c
13	Octanal	y = 0.2946x + 0.0373	0.9842	13.16 ± 0.24e	138.49 ± 2.98c	150.21 ± 2.96b	155.72 ± 8.35ab	161.79 ± 2.83a	159.96 ± 3.20a	131.08 ± 2.25c	66.98 ± 1.39d
14	Heptanal	y = 0.2338x + 0.0164	0.9699	10.02 ± 0.02d	115.33 ± 7.37c	120.75 ± 6.71c	137.88 ± 3.06ab	143.28 ± 3.94a	137.45 ± 5.47a	131.79 ± 3.14b	112.10 ± 2.14c
15	Benzaldehyde	—	—	ND	11.69 ± 1.06d	19.07 ± 2.09c	16.95 ± 1.72cd	19.90 ± 3.08c	115.32 ± 4.95b	120.18 ± 3.28ab	121.23 ± 1.75a
16	Nonanal	y = 0.3696x + 0.1004	0.9825	13.21 ± 0.45g	113.76 ± 4.96f	226.13 ± 7.67d	222.06 ± 10.86d	242.08 ± 4.90c	354.57 ± 5.84a	297.53 ± 5.75b	139.83 ± 2.35e
17	Pentanal	y = 0.0627x + 0.0241	0.9864	ND	23.69 ± 1.92d	125.04 ± 4.66a	122.51 ± 1.69a	120.53 ± 3.58a	121.23 ± 7.27a	109.66 ± 1.96b	55.08 ± 1.88c
18	Acetic acid	—	—	3.05 ± 0.08e	8.06 ± 0.89d	15.17 ± 1.36c	18.39 ± 0.66b	19.00 ± 0.53b	16.54 ± 3.10bc	23.59 ± 0.99a	25.17 ± 1.43a
19	Hexanoic acid	—	—	4.81 ± 0.12d	5.79 ± 0.32c	3.20 ± 0.20e	6.16 ± 0.43c	5.73 ± 0.28c	5.62 ± 0.49c	7.58 ± 0.21b	9.90 ± 0.42a
20	Decanoic acid	—	—	0.56 ± 0.05bc	0.58 ± 0.13bc	0.37 ± 0.09d	0.94 ± 0.07a	0.35 ± 0.02d	0.64 ± 0.14bc	0.69 ± 0.01b	0.51 ± 0.03cd
21	Nonanoic acid	—	—	0.92 ± 0.06c	0.81 ± 0.16c	0.60 ± 0.08d	0.84 ± 0.04c	0.81 ± 0.02c	1.34 ± 0.12a	1.11 ± 0.10b	0.86 ± 0.04c
22	Octanoic acid	—	—	1.28 ± 0.13a	0.71 ± 0.02c	ND	1.01 ± 0.10b	0.76 ± 0.76c	0.78 ± 0.78c	ND	0.94 ± 0.09b
23	Hexanoic acid methyl ester	—	—	14.32 ± 0.67a	ND	ND	ND	ND	ND	ND	ND
24	6-Methyl-5-hepten-2-one	—	—	10.52 ± 0.06c	11.20 ± 0.88bc	11.74 ± 0.01^bc^	11.58 ± 0.82bc	11.65 ± 0.34bc	11.97 ± 0.53b	14.41 ± 1.24a	14.61 ± 0.61a
25	2-Pentylfuran	y = 0.5325x + 0.0186	0.9749	1.55 ± 0.09e	1.60 ± 0.31e	31.89 ± 2.80^b^	22.25 ± 1.05c	36.33 ± 1.95a	34.58 ± 0.96ab	17.44 ± 1.66d	19.86 ± 1.77cd
26	2-Methypyrazine	y = 0.9420x + 0.0125	0.9683	ND	ND	ND	ND	11.33 ± 1.06d	53.28 ± 1.13c	77.54 ± 2.23b	101.70 ± 3.09a

The results were presented as means and standard errors. ND, not detected.

In all stages, hexanal, heptanal, (*E*)-2-octenal, (*E*)-2-heptenal, nonanal, and octanal were the most important aldehydes, and hexanal had the highest concentration. 1-octen-3-ol was the most important alcohol in roasted Tan mutton. The concentrations of most aldehydes and alcohols rose dramatically in roasted Tan mutton after 2–10 min of roasting (*P* < 0.05), but reduced after 12–14 min of roasting. In particular, hexanal (658.11 μg/kg), nonanal (354.57 μg/kg), heptanal (137.45 μg/kg), pentanal (121.23 μg/kg), octanal (159.96 μg/kg) and 1-octen-3-ol (320.31 μg/kg) may significantly contribute to the aroma of roasted Tan mutton after 10 min.

### Key aroma compounds in roasted Tan mutton

**Figure 2:**
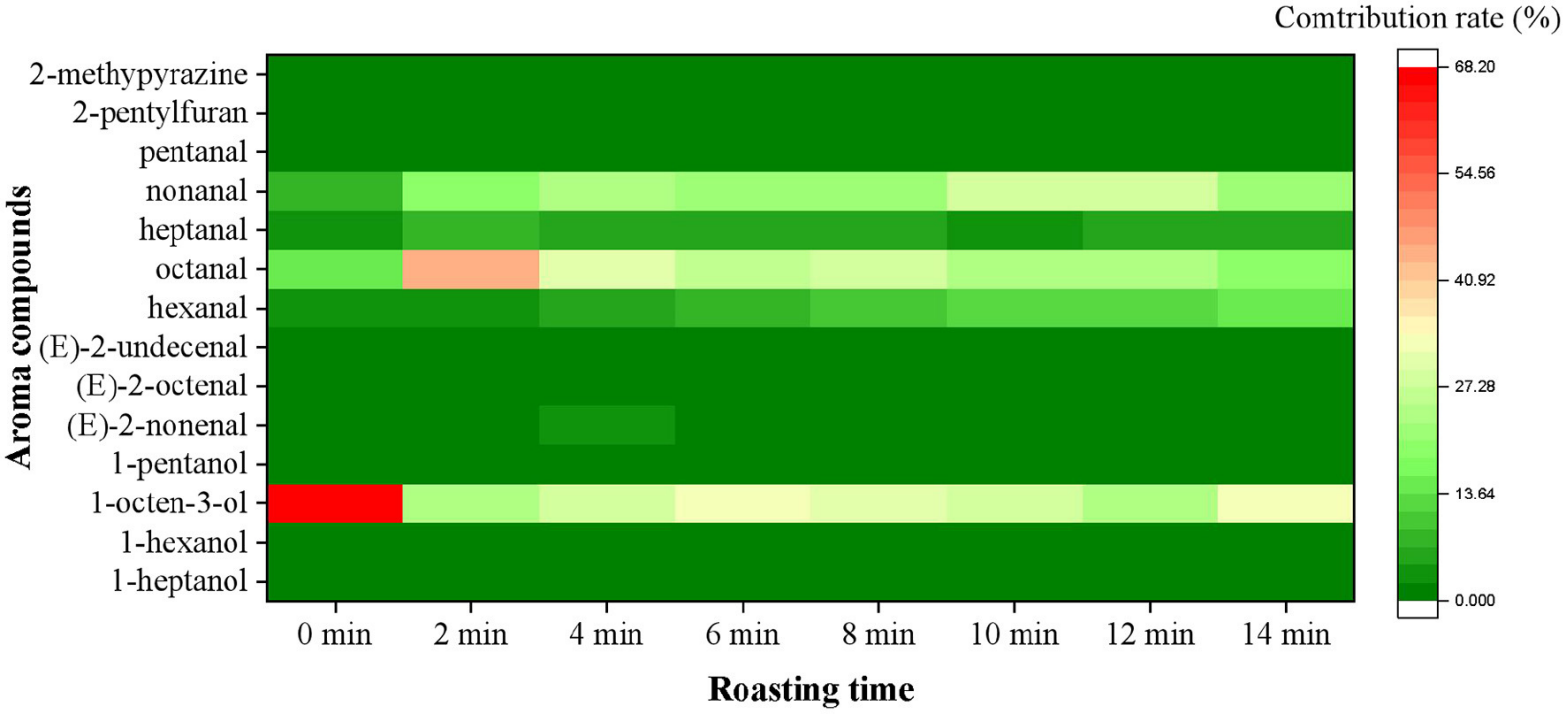
Changes of contribution rates of aroma compounds (OAV > 1) in roasted Tan mutton during the roasting process.

**Figure 3:**
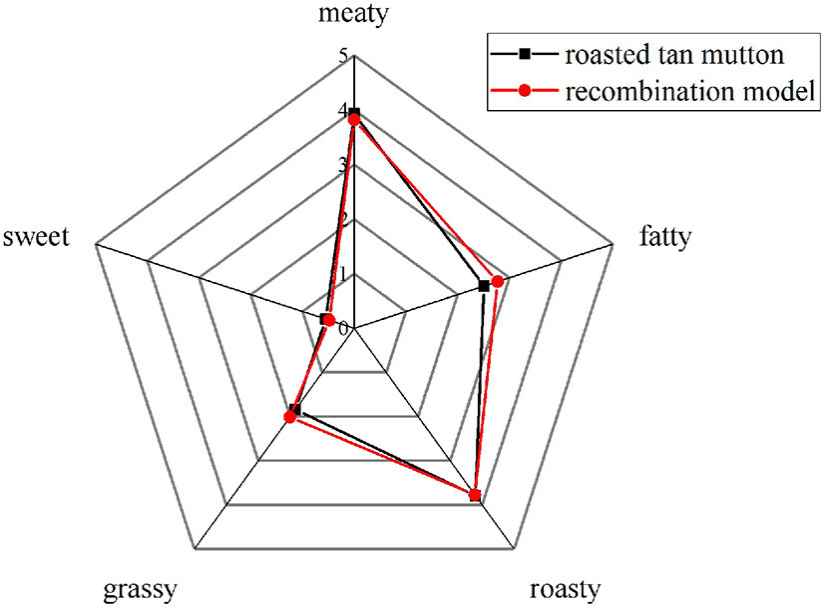
Aroma profiles in roasted Tan mutton and recombination model.

The OAV and contribution rate were calculated to better understand the significance of each aroma compound. As shown in Figure 2, a total of 14 aroma compounds, including (*E*)-2-octenal, 1-heptanol, hexanal, 1-hexanol, heptanal, 1-octen-3-ol, 1-pentanol, (*E*)-2-nonenal, octanal, (*E*)-2-undecenal, nonanal, pentanal, 2-pentylfuran and 2-methypyrazine were initially found as the key aroma compounds in roasted Tan mutton because their OAVs surpassed 1. Among them, there were eight aldehydes, four alcohols, one pyrazine and one furan. The concentrations and OAV of 14 key aroma compounds rose considerably (*P* < 0.05) from 0 to 10 min, but significantly decreased (*P* < 0.05) from 10 to 14 min. Only 7 of the 14 key aroma compounds with OAV greater than 1 may play critical roles in aroma expression in raw meat, including 1-hexanol, 1-octen-3-ol, (*E*)-2-nonenal, hexanal, octanal, heptanal, and nonanal. In comparison with raw meat, 14 key odorants were all observed and remained at high levels in the samples for 10 min, among which hexanal (146.24), nonanal (322.33), octanal (271.11), and 1-octen-3-ol (320.31) had the highest OAV. Particularly, the concentration and OAV of nonanal exhibited the highest level in the mutton roasted for 10 min. The contribution rate was further used to demonstrate the importance of each aroma compound. The nonanal (27.74%), 1-octen-3-ol (27.57%), octanal (23.34%), and hexanal (12.59%) primarily contributed to the aroma of roasted mutton for 10 min. Furthermore, the sensory panelists unanimously agreed that the recombination model of 14 key aroma compounds generated the usual meaty, grassy, roasty, fatty, and sweet aromas associated with the roasted mutton (Figure 3). The recombination model's similarity was rated 4.8 out of 5, indicating that the 14 aroma compounds were the key aroma compounds of roasted Tan mutton.

### Change of protein secondary structure in roasted Tan mutton

**Figure 4:**
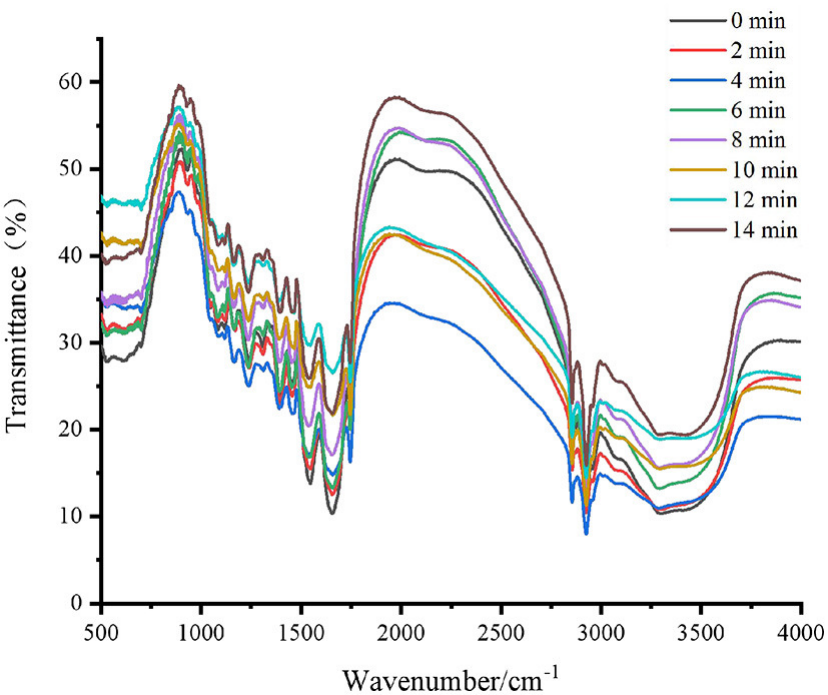
Fourier transform infrared spectroscopy (500–4000 cm^−1^) from roasted Tan mutton at different roasting time.

FT-IR spectroscopy is a commonly used and reliable method in the analysis of protein secondary structure. Our second-derivative band placements were consistent with previous studies showing that mid-IR spectra in the range of 1645–1662 cm^−1^ accounted for the α-helix band, the β-sheet band located at 1612–1640 cm^−1^ and 1682–1697 cm^−1^, the β-turn band located at 1662–1682 cm^−1^, and the random coil located at 1637–1645 cm^−1^ (32, 33). Fourier self-deconvolution, second derivative, and Gaussian curve-fitting were used in this investigation to quantitatively examine the spectra's second derivative (34). As shown in Table 3 and Figure 4, there was a reduction in the α-helix content (*P* < 0.05), and an increase in the β-sheet (*P* < 0.05) across all of the samples. The content of random coils significantly increased in the samples roasted for 0–8 min (*P* < 0.05), but afterward, no discernible change was found. At the same time, there were no significant changes in the content of β-turn. The contents of α-helix and β-sheet of protein from roasted Tan mutton decreased, and the contents of β-turn and random coil increased. Higher levels of α-helix and β-sheet indicated a more stable secondary structure, whereas higher contents of β-turn and random coil indicated a more flexible protein structure (35).

**Table 3:** Relative percentage of protein secondary structure of roasted Tan mutton.

	**0 min**	**2 min**	**4 min**	**6 min**	**8 min**	**10 min**	**12 min**	**14 min**
α-helix (%)	37.66 ± 0.27a	35.23 ± 0.25b	33.05 ± 0.12c	28.20 ± 0.41d	26.81 ± 0.39e	26.55 ± 0.14e	24.85 ± 0.07f	24.43 ± 0.16f
β-sheet (%)	17.13 ± 0.10f	19.70 ± 0.40e	20.67 ± 0.50d	22.69 ± 0.45c	23.16 ± 0.20c	25.67 ± 0.35b	26.11 ± 0.16ab	26.46 ± 0.21a
β-turn (%)	13.69 ± 0.31a	12.48 ± 0.20d	12.22 ± 0.36d	12.65 ± 0.24cd	13.08 ± 0.14bc	13.49 ± 0.20ab	13.20 ± 0.17ab	13.56 ± 0.33ab
random coil (%)	31.52 ± 0.27e	32.59 ± 0.52d	34.06 ± 0.33c	36.48 ± 0.30a	36.95 ± 0.08a	34.29 ± 0.22c	35.84 ± 0.06b	35.55 ± 0.32b

## Discussion

### Aldehydes and alcohols are the pivotal aroma compounds in roasted Tan mutton

The pivotal aroma compounds in meat were aldehydes and alcohols, such as hexanal, nonanal, octanal, and 1-octen-3-ol (36). In this study, 12 aldehydes and alcohols were identified out of 14 key aroma compounds, with a percentage contribution of 98.91–99.95% in the aroma of roasted Tan mutton. Nonanal had the highest OAV (322.34) and contribution rate (27.74%) especially, followed by 1-octen-3-ol, octanal and hexanal in the mutton roasted for 10 min. According to the investigation, lipid oxidation products, such as 1-octen-3-ol, heptanal, hexanal and octanal, had the highest concentrations and OAV in the roasted mutton (4, 13, 37). Although the concentrations of aldehydes and alcohols varied depending on the roasting procedure, these compounds remained the most prominent aroma compounds in the roasted mutton (11).

**Figure 5:**
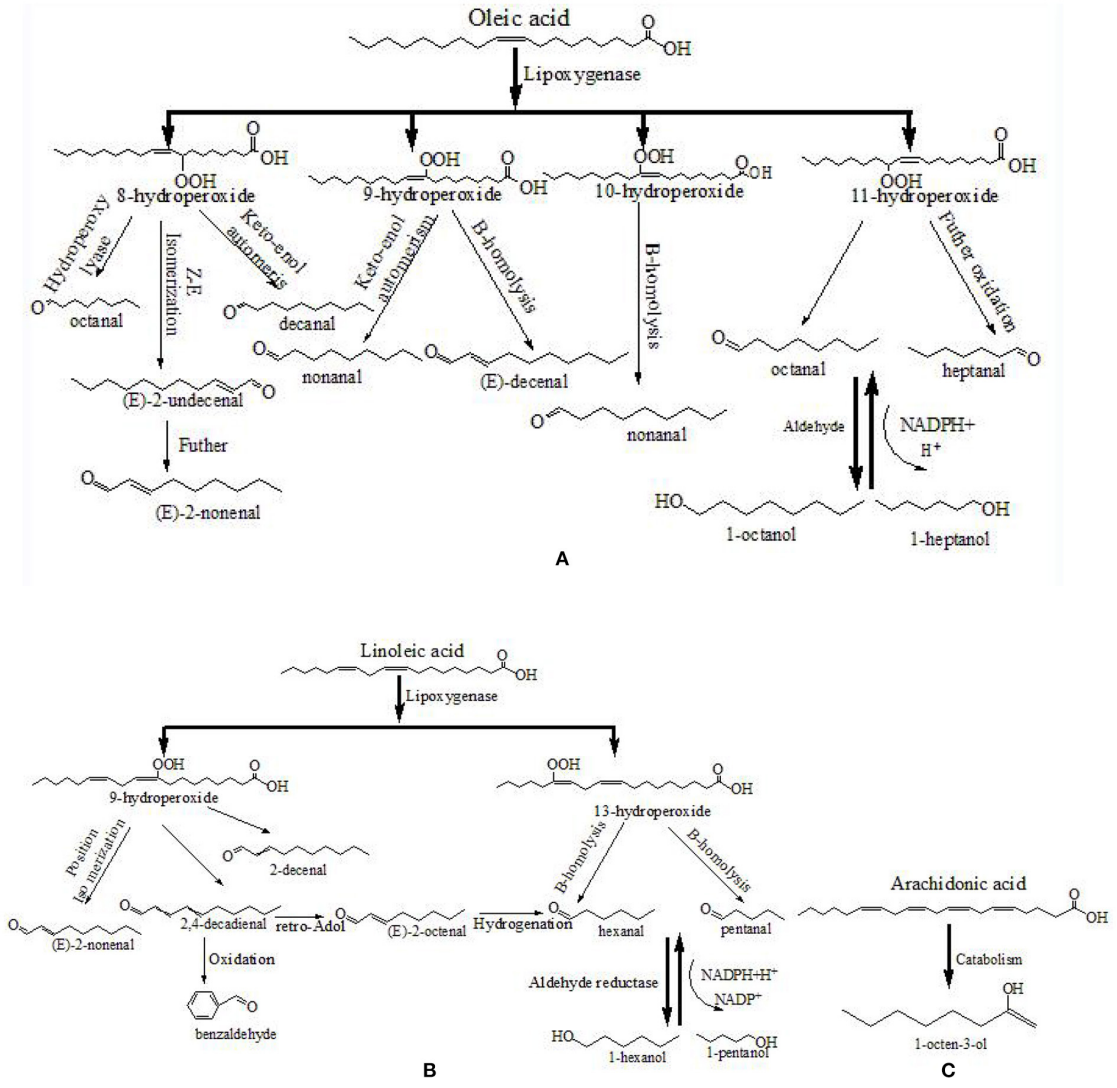
Formation pathway of the typical aliphatic aldehydes from the fatty acid **(A–C)**. Referred to Al-Dalali et al. (38).

Unsaturated fatty acids were the primary contributors to the formation of fatty aldehydes and alcohols. In various livestock and poultry meat, oleic acid is the common monounsaturated fatty acid, while linoleic acid, linolenic acid, and arachidonic acid are the common polyunsaturated fatty acids (8). Figure 5 depicts the formation pathway of some aliphatic aldehydes and alcohols from fatty acid degradation. First, hydroperoxides of fatty acids are formed by removing the hydrogen-free radical from the alkyl radical, adding O_2_, and absorbing the hydrogen-free radical (39). The hydroperoxides are then fractured, which results in the generation of volatile chemicals. As shown in Figure 5, the decomposition of oleic acid 8- and 11-hydroperoxides may result in the formation of octanal and decanal, respectively (40). The decomposition of linoleic acid 9- and 13-hydroperoxides may result in the formation of (*E, E*)-2,4-decadienal and hexanal, respectively (38, 41). Furthermore, the generated aldehydes can undergo further reactions, such as alcohol or acid transformation or retro-aldol condensation (9, 42). The decomposition of 9- and 13-hydroperoxides of linoleic acid can produce (*E, E*)-2,4-decadienal, and the retro-aldolization of (*E, E*)-2,4-decadienal can result in the formation of (*E*)-2-octenal.

As for the 14 key aroma compounds in roasted Tan mutton, the major source of 1-octen-3-ol, which has a mushroom odor, is arachidonic acid (38, 43). Hexanal, pentanal, heptanal, (*E*)-2-nonenal, (*E*)-2-octenal, 1-pentanol and 2-pentylfuran are derived from the oxidized linoleic acid, while octanal, 1-hexanol, 1-heptanol nonanal and 2-undecenal are mainly oxidized from oleic acid. Furthermore, 2-methypyrazine may also be generated from lipid-Maillard interaction and a higher pH value contributed to its generation (44, 45).

### Protein secondary structure in roasted Tan mutton

Protein is a key quality indicator in the roasted meat, influencing the color, texture and flavor of the meat (46–48). Particularly, some physiologically active enzymes in meat have a direct impact on meat tenderness (49). The amide I band is the most useful band to analyze secondary structural information of proteins. The alterations in the FTIR band in the current investigation were in accordance with previous studies showing that the level of β-sheet gradually increased and the α-helix content decreased during roasting process (50, 51). The decrease of α-helix content suggested that myofibrillar proteins were uncoiling and nonpolar amino acids were exposed to the surface of proteins in roasted Tan mutton (51, 52). The exposure to nonpolar amino acids led to the improvement of surface hydrophobicity, and this improvement was sensitive to the change in roasting temperature (53). The interaction between nonpolar amino acids was greatly strengthened, leading to protein aggregation when roasted at high temperatures (54). Protein aggregation significantly altered the structure of proteins, and β-sheet was shown to be intimately associated with protein aggregation (55). The increase of β-sheet content in roasted Tan mutton during the roasting process may be due to the effects of heating on the reconstruction of unfolded myofibrillar proteins and the aggregation of myofibrillar proteins *via* hydrophobic interaction between nonpolar amino acids (56, 57). Interestingly, an increased content of random coils was observed in the samples roasted for 0–8 min, suggesting that random coils were formed as a result of extreme denaturation of myofibrillar proteins (58).

### Relationship between protein secondary structure and key aroma compounds

Many flavor compounds, including ketones, aldehydes and esters, bind to proteins *via* hydrophobic interactions (59–61). During the roasting process, a high energy input may cause a significant degree of denaturation and destruction of the secondary structure (62, 63). Changes in the microstructure of macromolecules, such as changes in protein conformation (α-helix, β-sheet, and β-turn), can alter the interaction between volatile chemicals and proteins (64). Finally, the phase equilibrium of the flavor compound system may be broken.

**Figure 6:**
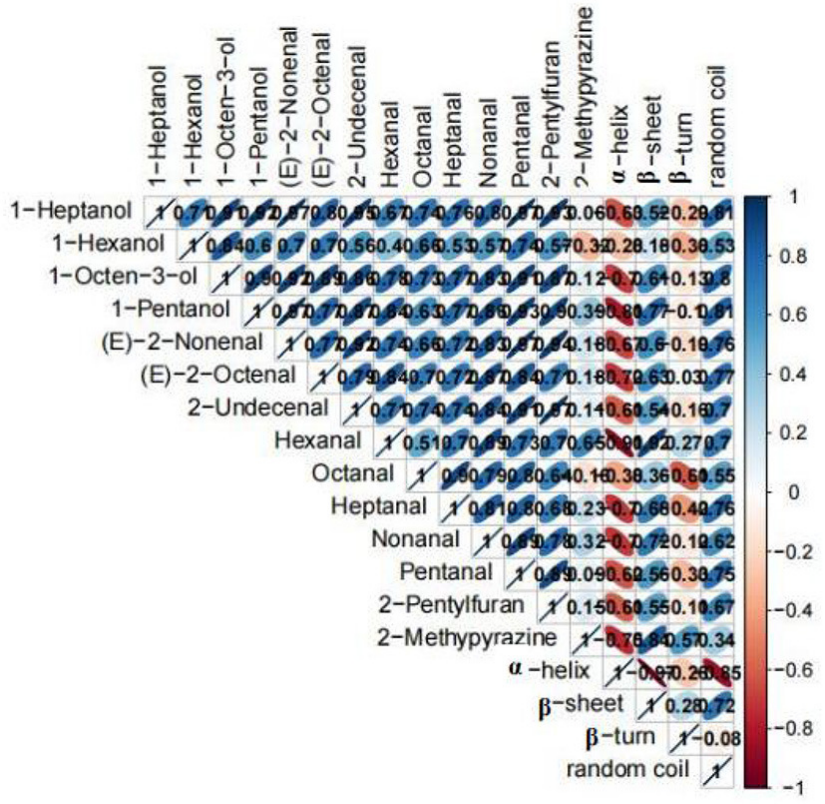
Correlation matrix between parameters of proteins structures (α-helix, β-sheet, β-turn, and random coil) and key aroma compounds.

To understand the relationship between the structure of proteins and the key aroma compounds during the roasting process, the Pearson correlation analysis was performed (Figure 6). The 14 key aroma compounds were all positively correlated with α-helix and were all negatively correlated with β-sheet and random coil in the amide I band and were also positively correlated with β-turn except hexanal and 2-methypyrazine. Among them, hexanal both had the highest correlation coefficient with α-helix and β-sheet.

## Conclusion

Thermal processing methods have a significant influence on the aroma of mutton. In this study, 14 aroma compounds, including 1-heptanol, 1-hexanol, 1-octen-3-ol, 1-pentanol, (*E*)-2-nonenal (*E*)-2-octenal, (*E*)-2-undecenal, hexanal, octanal, heptanal, nonanal, pentanal, 2-pentylfuran, and 2-methypyrazine, were identified as key aroma compounds in roasted Tan mutton under charcoal grilling conditions. Among them, the key aroma compounds were aldehydes and alcohols. A reduction in the α-helix content (*p* < 0.05) and an increase in the β-sheet level (*p* < 0.05) were observed during the whole roasting process. Furthermore, random coil content significantly increased in the samples roasted for 0–8 min and β-turn content did not change. Correlation analysis showed that 14 key aroma compounds were all positively correlated with α-helix and negatively correlated with β-sheet and random coil in the amide I band, and were also positively correlated with β-turn except hexanal and 2-methypyrazine. In the next step, we will study the key volatile compounds of common condiments to lay the foundation for revealing the interaction between the aroma of roasted Tan mutton and condiments, which is helpful to promoting the industrialization of roasted Tan mutton.

## Data availability statement

The original contributions presented in the study are included in the article/supplementary material, further inquiries can be directed to the corresponding author.

## Author contributions

Y-RW: conceptualization, methodology, software, data curation, and writing—original draft preparation. S-LW: visualization and investigation. R-ML: software, validation, and writing—reviewing and editing. All authors contributed to the article and approved the submitted version.

## Funding

The State Key Research and Development Plan (2018YFD0400101), the Natural Science Foundation of China (31660484), and the Key Research and Development Plan of Ningxia Hui Autonomous Region (2019BEH03002) provided financial assistance for this work.

## Conflict of interest

The authors declare that the research was conducted in the absence of any commercial or financial relationships that could be construed as a potential conflict of interest.

## Publisher's note

All claims expressed in this article are solely those of the authors and do not necessarily represent those of their affiliated organizations, or those of the publisher, the editors and the reviewers. Any product that may be evaluated in this article, or claim that may be made by its manufacturer, is not guaranteed or endorsed by the publisher.
